# Imaging and Resuscitation in Trauma

**DOI:** 10.1155/2011/313404

**Published:** 2011-12-26

**Authors:** Aristomenis K. Exadaktylos, Marc A. de Moya, Fiona Lecky, Peter Driscoll, Heinz Zimmermann, Lee Alan Wallis, Robert A. Novelline

**Affiliations:** ^1^Department of Emergency Medicine, Inselspital, University of Bern, 3010 Bern, Switzerland; ^2^Massachusetts General Hospital-Harvard University, Boston, MA 02114, USA; ^3^University of Manchester and Manchester Medical Academic Health Sciences Center, Manchester M139PL, UK; ^4^Salford Royal Hospitals NHS Foundation Trust, University Hospital, University of Manchester, Salford M68HD, UK; ^5^Division of Emergency Medicine, Stellenbosch University and University of Cape Town, Provincial Government of the Western Cape, Stellenbosch 7602, South Africa; ^6^Radiology Department, Harvard Medical School, Boston, MA 02114, USA

Injury is expected to become the second leading cause of death by 2020 [1]. 

The initial evaluation of a critically injured polytrauma patient is a challenging task and every minute can make the difference between life and death. It has been shown that mortality decreases as the time from injury to diagnosis and treatment is shortened [1]. Over the past few years, the assessment of trauma patients has evolved, due to the improved understanding of the mechanisms that contribute to morbidity and mortality in trauma, and this has led to the development of advanced trauma life support. 

Because hemorrhagic shock is the second leading cause of death after trauma, the optimal treatment for bleeding has become a major priority. In their paper, V. Jeger et al. “The role of thromboelastography in multiple trauma” describe thrombelastography (TEG) as a very useful tool in managing bleeding patients, and we suspect that this procedure will become a gold standard of resuscitation during the next decade. Since fluid resuscitation is one of the major tasks in the management and treatment of severely burned patients, the paper by M. Stander et al. “The emergency management and treatment of severe burns” is really significant, as it presents a simple guideline for the initial management of severe burns, as utilised by the South African Burn Society.

But what would treatment be without imaging? Quick and cost-effective imaging has become one of the cornerstones of modern trauma management. R. Kaewlai et al. “Blunt cardiac injury in trauma patients with thoracic aortic injury” and P. D. Levy et al. “Micropower impulse radar: a novel technology for rapid, real-time detection of pneumothorax” present some very promising data on detecting injuries of the heart and lungs. Micropower impulse radar, for example, is a novel technology for the rapid detection of pneumothorax, with the power to simplify decision making in the prehospital sector as well as in the trauma bay and to reduce the burden of radiation for patients and staff. Since no technology is perfect and any 100% sensitivity and specificity margin for diagnosing injuries is a myth, we focus on the importance of the followup of trauma patients and raise the awareness for even rare but potentially life-threatening complications. 

*Aristomenis K. Exadaktylos* *Aristomenis K. Exadaktylos* *Marc A. de Moya* *Marc A. de Moya* *Fiona Lecky* *Fiona Lecky* *Peter Driscoll* *Peter Driscoll* *Heinz Zimmermann* *Heinz Zimmermann* *Lee Alan Wallis* *Lee Alan Wallis* *Robert A. Novelline* *Robert A. Novelline*
